# Improved Bacterial Mutagenesis by High-Frequency Allele Exchange, Demonstrated in Clostridium difficile and Streptococcus suis

**DOI:** 10.1128/AEM.01195-13

**Published:** 2013-08

**Authors:** Alexandra Faulds-Pain, Brendan W. Wren

**Affiliations:** Department of Pathogen Molecular Biology, London School of Hygiene and Tropical Medicine, London, United Kingdom

## Abstract

Here we show that the frequency of mutant isolation by two-step allele exchange can be improved by increasing the length of homologous DNA and the opportunity for recombination, obviating the need for counterselection markers. These principles are demonstrated in Clostridium difficile and Streptococcus suis but are likely to be generally applicable.

## TEXT

**Fig 1:**
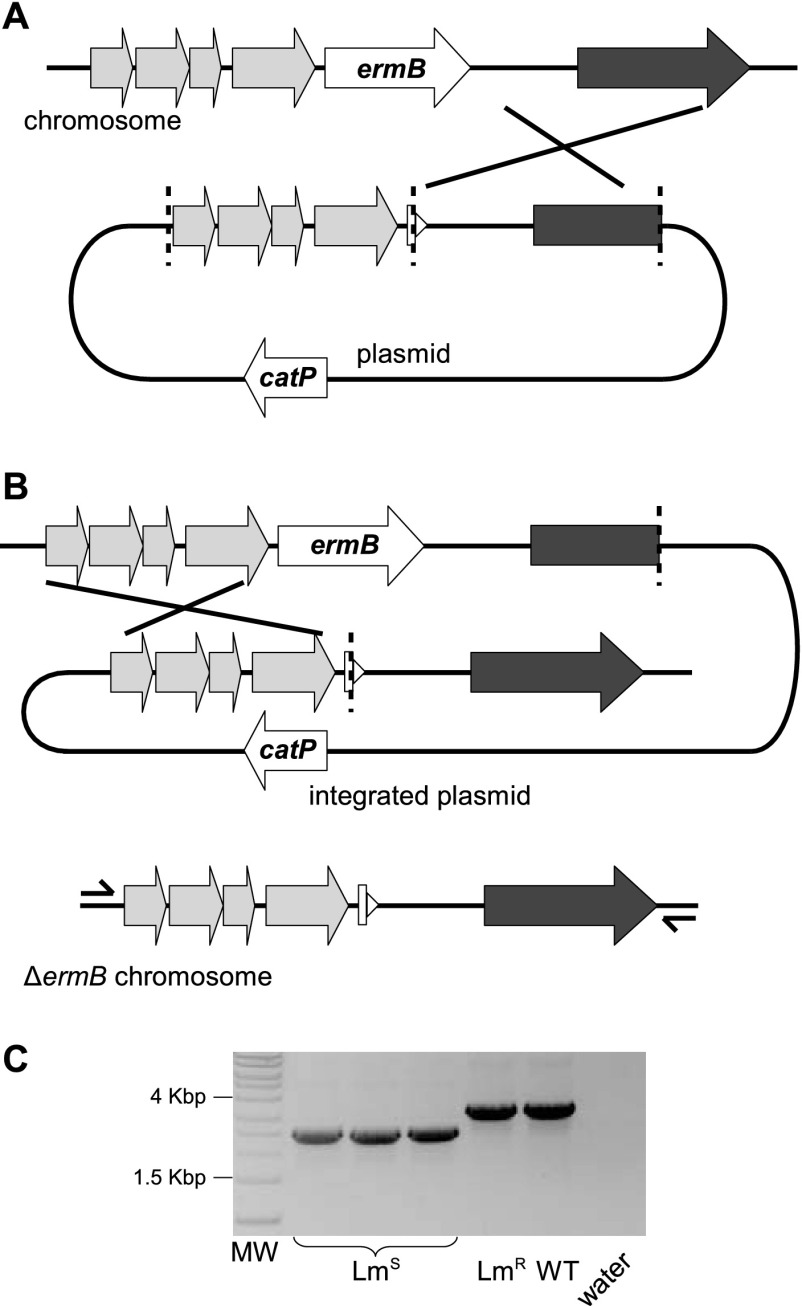
Diagrammatic representation of the precise deletion of *ermB* in C. difficile strain M68 by two-step allele exchange. (A) The first recombination event between the pAFP1200 plasmid and the *ermB* locus. In this example, integration at the right region of homology (dark gray) is represented. (B) The second recombination event causes a deletion of the *ermB* gene; if the recombination event occurs at the other region of homology to the first recombination event (light gray), the gene is deleted from the chromosome. (C) PCR screening confirms the *ermB* deletion mutants initially identified by replica plating on thiamphenicol (Tm), lincomycin (Lm), and nonselective media. PCR was performed using primers M68Δ*ermB* screen F and M68Δ*ermB* screen R (see Table S2 in the supplemental material). MW, Hyperladder I molecular weight marker; Lm^S^, DNA from Lm-sensitive clones; Lm^R^, DNA from Lm-resistant but Tm-sensitive clone; WT, wild-type DNA; water, water-only control.

Mutagenesis is central to the study of microorganisms, enabling the functional role of specific genes to be determined (1, 2). There are many important organisms for which reliable allele exchange methods are not well established, including several pathogens. Allele exchange, or gene replacement, is perhaps the most widely implemented strategy of directed mutagenesis in microorganisms whereby a native allele is exchanged with an introduced, alternative allele containing a mutation. The alternative allele is flanked by DNA regions identical to the DNA regions flanking the original allele (Fig. 1A). Homologous recombination events between both pairs of identical DNA regions are required to mediate the exchange. When replication-defective plasmids (suicide, conditional, or “pseudosuicide” [3–8]) are used as the delivery vehicle for the allele exchange cassette, mutagenesis is a two-step process. The first recombination event, termed a single-crossover event, causes integration of the plasmid into the genome and is simply isolated when positive, plasmid-specific antibiotic selection is applied. A second recombination event (a double-crossover event) can then occur whereby the plasmid is excised from the genome. If this second event occurs at the same region of homology as the first event, then the cell reverts to wild type; if it occurs at the other region of homology, then the alternative allele is stably localized, causing the desired mutation (Fig. 1B).

The isolation of double-crossover clones can be achieved by incorporating a counterselection marker onto the allele exchange plasmid whose presence causes cell death under appropriate conditions (3, 9–12). Identification of a suitable counterselection marker is complex and often organism specific (11); however, it is often assumed that isolating mutants using two-step allele exchange is extremely laborious without a counterselection marker due to the low frequency at which the second recombination event occurs (11). We questioned whether this assumption is justified and whether the frequency of recombination could be increased, in a generally applicable way, to a level that would make two-step allele exchange facile and obviate the requirement of a counterselection marker.

We undertook to increase the frequency of the second recombination event in two ways. The first was by using long regions of homologous DNA which we hypothesized would increase the frequency of the second recombination event (5). The second was to increase the opportunity for recombination to occur by serially subculturing single-crossover integrants. This second principle is an advantage particular to two-step allele exchange, as members of a population of single-crossover clones all have the potential to undergo a second recombination and become double crossovers, and this potential is not lost until a recombination event occurs.

Clostridium difficile is the main cause of antibiotic-associated diarrhea in health care settings. There are at least five clonal lineages of C. difficile, designated clades 1 to 5 (13–17), but to date, only strains from clades 1 and 2 have been studied by a directed mutagenesis approach. The most widely implemented method of C. difficile mutagenesis introduces the macrolide, lincosamide, streptagramin (MLS) antibiotic resistance marker *ermB* into MLS-sensitive strains (6, 7). We are interested in studying isogenic mutants of clinical isolates from clade 4 using this approach (13, 17). However, we found that strains in this clade were resistant to the MLS antibiotic lincomycin (Lm) (see Table S1 and Materials and Methods in the supplemental material) and that this was likely to be due to the presence of a chromosomal *ermB* gene (14, 18). Therefore, our first target for our alternative approach to allele exchange was the *ermB* gene in the clade 4 sequenced strain M68.

The allele exchange cassette for the deletion of *ermB* from M68 was constructed by SOE-PCR (19) with 1,200-bp regions of DNA homologous to the DNA sequences flanking the *ermB* gene (Fig. 1A) using a high-fidelity polymerase (Phusion; NEB). This was cloned into the replication-defective C. difficile vector pMTL82151 (20), suitable for pseudosuicide in C. difficile (4, 5), using standard cloning techniques, resulting in the plasmid pAFP1200 (see Table S2 in the supplemental material for primers and restriction enzymes). pAFP1200 was transferred into C. difficile M68 by conjugation (4, 7, 21), and single-crossover clones were obtained by passage three times on selective media as described previously (3, 4) (Fig. 1B). C. difficile was cultured routinely at 37°C in an anaerobic work station (Don Whitley) on BHIS medium (brain heart infusion [BHI] medium supplemented with 5 mg · ml^−1^ yeast extract and 0.1% [wt/vol] l-cysteine) supplemented with d-cycloserine (250 μg · ml^−1^), cefoxatin (8 μg · ml^−1^), and thiamphenicol (Tm) (15 μg · ml^−1^) to maintain plasmid selection.

In the second part of the experiment, thiamphenicol selection was removed to allow growth of double-crossover clones. To allow more opportunity for the second recombination event to occur, the single-crossover clones were serially subcultured without selection for up to 9 consecutive days. Double-crossover recombinants were identified by replica plating colonies onto nonselective BHIS and BHIS media with thiamphenicol to screen for the loss of the plasmid-specific antibiotic resistance marker, *catP*. As the target gene, *ermB*, is likely to confer resistance to lincomycin, deletion mutants could also be distinguished from revertants by replica plating on lincomycin (40 μg · ml^−1^). We found that following subculture, thiamphenicol-sensitive clones (Tm^S^; recombinants) that were either lincomycin sensitive (Lm^s^; deletion mutants) or lincomycin resistant (Lm^r^; revertants) were isolated. By the fifth nonselective subculture, 1 clone of the 50 replica-plated clones was Tm^s^ Lm^s^ and one clone Tm^s^ Lm^r^, indicating that one mutant and one wild-type revertant had been isolated; by passage six, 8 of the 75 replica-plated clones were Tm^s^ Lm^s^ and two were Tm^s^ Lm^r^. The Δ*ermB* genotype was confirmed by PCR (Fig. 1C), and the products were sequenced (Source Bioscience). One clone (designated M68Δ*ermB*) was checked and found to be sensitive to lincomycin (MIC 32 μg · ml^−1^, while M68 was resistant at 128 μg · ml^−1^) and erythromycin (MIC 1 μg · ml^−1^, while M68 was resistant at 128 μg · ml^−1^).

The ease with which mutant clones were obtained in this experiment suggested that, as predicted, the frequency of the second recombination event is a function of the length of the homology region. To further determine the effect of the length of the homology region on the frequency of the second recombination event, two additional allele exchange plasmids were constructed to make the same deletion in M68 *ermB* with 300-bp and 600-bp regions of homology (pAFP300 and pAFP600, respectively) and conjugated into C. difficile M68.

**Fig 2:**
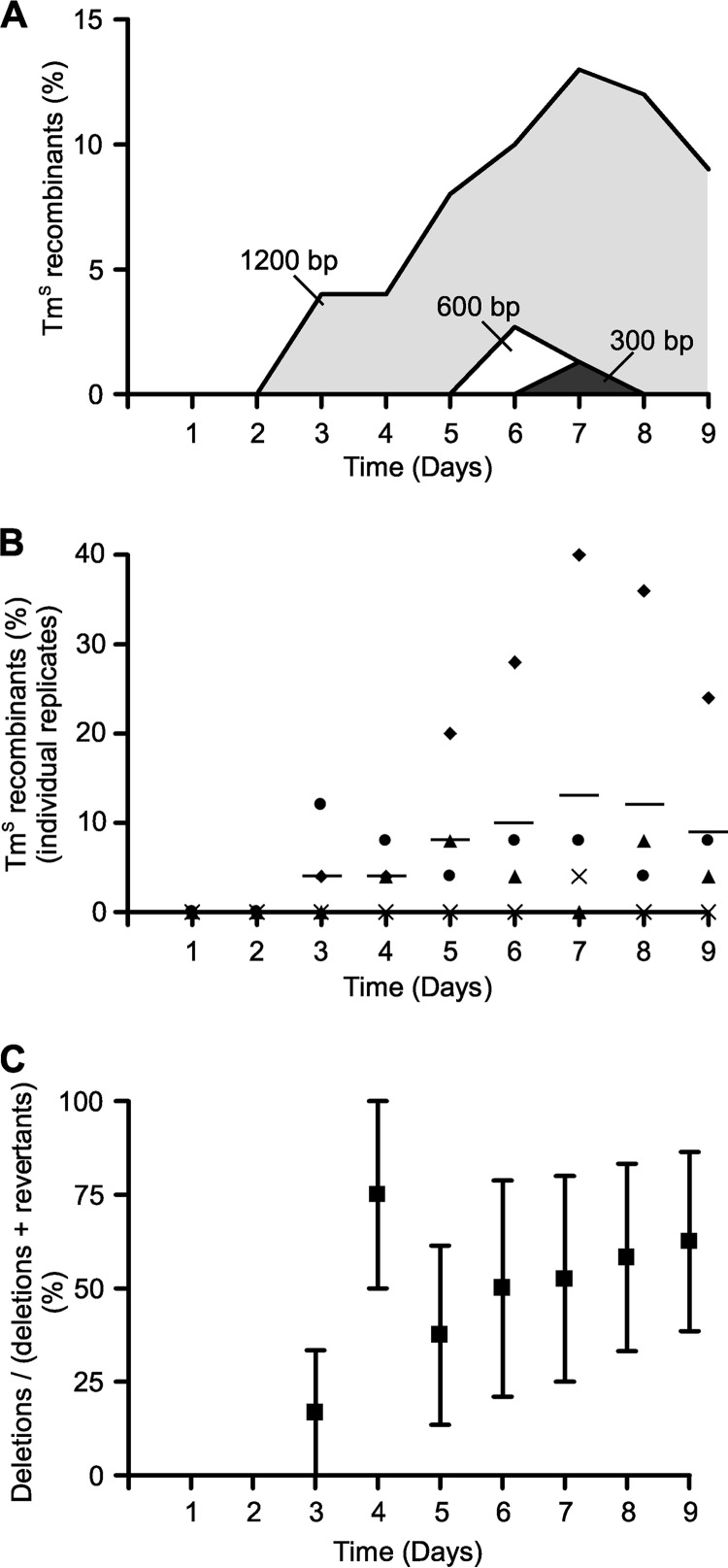
Recombination frequency and the importance of the lengths of the homology regions. (A) The graph represents the fraction of double-crossover recombinants as a percentage of the total clones screened. Single crossovers of the three *ermB* deletion plasmids with differing homology lengths (pAFP300, pAFP600, and pAFP1200) were passaged without selection and screened each day for loss of Tm resistance; the data plotted represent the average results of either three or four replicates, each of which screened 25 colonies a day. Light gray, 1,200-bp, double-crossover recombinants from pAFP1200 single-crossover recombinants; white, 600-bp, double-crossover recombinants from pAFP600 single-crossover recombinants; dark gray, 300-bp, double-crossover recombinants from pAFP300 single-crossover recombinants. (B) The graph represents the fraction of pAFP1200 single-crossover cells that had undergone a second recombination event and become Tm^s^ when passaged without selection every day for 9 days. Each of the symbols ▲, ●, ×, and ◆ represents a different replicate; the horizontal line represents the mean of the four repeats. (C) The graph represents the fraction of the pMTLAFP1200 double-recombinant clones (deletions plus revertants) that are deletions, as a percentage, over time. The large filled squares represent the average results of four repeat experiments where 25 clones were screened per repeat; error bars represent the standard errors of the means.

Single-crossover clones derived from pAFP300, pAFP600, and pAFP1200 were confirmed by PCR and serially subcultured without selection daily for 9 days. The empty vector pMTL82151 was used as a control. Each day, 25 colonies were replica plated to determine whether a second recombination event had occurred and, if so, whether this had resulted in reversion to the wild type or deletion of the *ermB* gene. Figure 2A shows the average frequency of Tm^s^ recombinants over time. We found that double-crossover clones occurred at a considerably higher frequency with 1,200-bp regions of homology than with either 300 bp or 600 bp. Although it was possible to isolate double-crossover clones using 300-bp or 600-bp regions of homology (Fig. 2A), neither was consistent. The second recombination event was not detected in every replicate for either 300-bp or 600-bp regions, and the frequency was altogether too low to make it a reliable approach to mutagenesis (one double crossover with 300 bp and three double crossovers with 600 bp of the 25 clones per subculture for nine subcultures in triplicate). Following a single subculture without selection, the empty plasmid was lost from the control strain.

Using pAFP1200, double-crossover clones tended to be isolated from the third or fourth subculture onward (Fig. 2A and B). The frequencies of the second recombination event differed between the four repeats (Fig. 2B), but on average, the frequency between days 3 and 9 remained between 4% and 13% of the total clones screened; therefore, double-crossover clones were readily isolated by replica plating 25 colonies. Deletion mutants and revertants were isolated at similar frequencies between days 3 and 9 (Fig. 2C).

**Fig 3:**
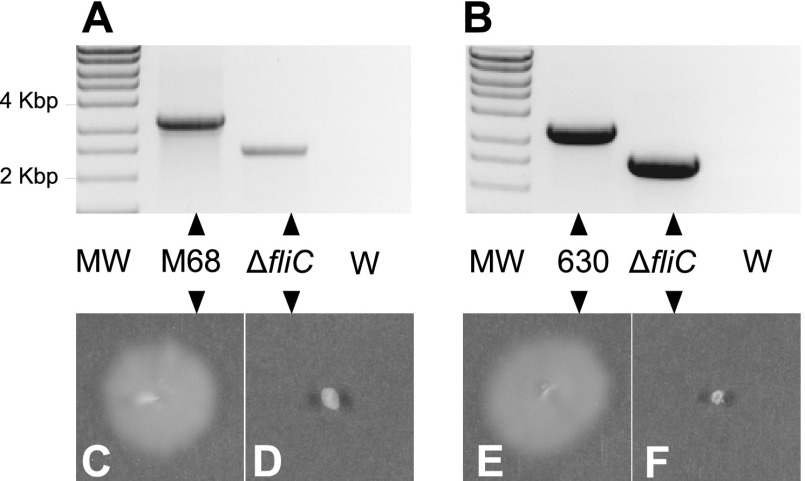
Deletion of the *fliC* gene encoding flagellin from strains M68 and 630 of C. difficile. (A) PCR screening spanning the *fliC* gene to confirm its deletion from M68 using primers M68 Δ*fliC* screenF and M68 Δ*fliC* screenR. (B) PCR screening spanning the *fliC* gene in the strain 630 and 630Δ*fliC* to confirm deletions. MW, Hyperladder I molecular weight marker; M68, M68 parental DNA; 630, 630 parental DNA; Δ*fliC*, *fliC* mutant DNA; W, water control. Motility assays in 0.3% agar of the C. difficile M68 wild type (C) and the Δ*fliC* mutant (D) showed that M68 is motile but that Δ*fliC* is nonmotile. Motility assays in 0.3% agar confirmed that the 630 strain is motile (E) and the Δ*fliC* mutant is nonmotile (F). Filled triangles indicate the gel lane to which the label relates, and the inverted filled triangles indicate the phenotypic verification by motility assays for these strains.

These results indicate that, using 1,200-bp regions of homology, the frequency of recombination is sufficient to make screening alone a reliable method of identifying double-crossover recombinants, a phenomenon that should be generally applicable to any nonessential open reading frame. We applied this principle to the deletion of *fliC* encoding flagellin from C. difficile. Allele exchange plasmids for the deletion of *fliC* from M68 (pAFP182) and the clade 1 strain, 630 (pAFP91), were constructed with 1,200-bp regions of homology and single-crossover integrants isolated. Following five nonselective subcultures, mutants were identified among double crossovers by PCR screening (Fig. 3A and B). One mutant of each strain (accordingly designated M68Δ*fliC* and 630Δ*fliC*) was found to be nonmotile in contrast to its parent strain in plate motility assays (25-ml plates of BHIS medium containing 0.3% Bacto agar [Difco] were inoculated with a single colony and incubated for 48 h and the images captured using a Cannon 600D digital camera) (Fig. 3C, D, E, and F).

The usefulness of long regions of homology and serial subculturing to facilitate mutagenesis seems unlikely to be unique to C. difficile. Streptococcus suis serotype 2 is a major pathogen of swine that has recently been reported to have crossed the species barrier, causing infections in humans (22, 23). The few reports to date of mutagenesis of S. suis used allele exchange without a counterselection marker and necessitated the laborious screening of large numbers of colonies (24–26). A major virulence determinant of S. suis is its polysaccharide capsule, and the *cps2E* gene is thought to be essential for capsule formation. In order to generate a Δ*cps2E* mutant for further study, the high-frequency approach to allele exchange was applied.

**Fig 4:**
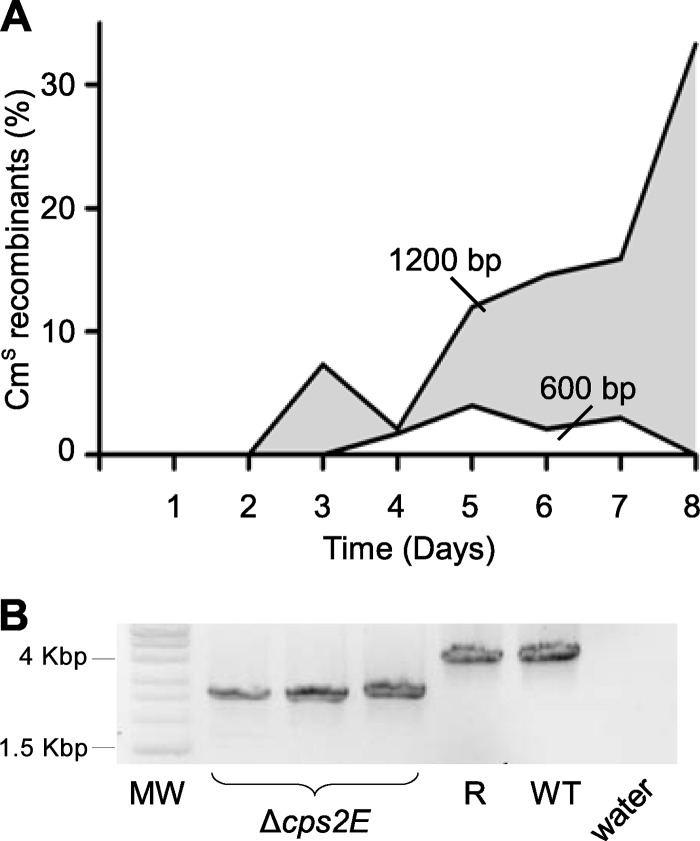
Deletion of the *cps2E* gene from S. suis P1/7 and the role of the length of the homology region in the frequency of recombination. (A) The graph represents the fraction of double-crossover recombinants as a percentage of the total clones screened. Single crossovers of two *cps2E* deletion plasmids, one with 600-bp and the other with 1,200-bp regions of homology (pAFP211 and pAFP212, respectively), were passaged without selection and screened for loss of chloramphenicol resistance. The data plotted represent the average results of three replicate experiments. Gray, 1,200-bp, double-crossover recombinants from pAFP212; white, 600-bp, double-crossover recombinants from pAFP211 single-crossover clones. (B) PCR screening spanning the *cps2E* gene in S. suis P1/7 to identify deletions from revertants. MW, Hyperladder I molecular weight marker; Δ*cps2E*, *csp2E* deletion mutant DNA; R, revertant to wild-type DNA; WT, wild-type P1/7 DNA; water, water control. Primers *cps2E*screen F and *cps2E*screen R were used and produced bands of predicted sizes, for parental DNA and mutant DNA, of 4,011 bp and 2,667 bp, respectively.

First, we determined that pMTL82151 was nonreplicative in S. suis (see the supplemental material). Two allele exchange cassettes for the deletion of *cps2E*, one with 600-bp regions of homology (pAFP211) and the other with 1,200-bp regions of homology (pAFP212), were then constructed. S. suis P1/7 was cultured at 37°C in a 5% CO_2_ incubator and grown on BHI medium supplemented with chloramphenicol (5 μg · ml^−1^) where appropriate. Transformation of the allele exchange plasmids by electroporation was done as previously described (27), and chloramphenicol-resistant single-crossover clones were obtained. Single crossovers were passaged daily without selection, and each day, colonies were replica plated onto chloramphenicol and nonselective agar to determine whether the second recombination event had occurred. As with C. difficile, the frequency of the second recombination was considerably higher with 1,200-bp than with 600-bp regions of homology (Fig. 4A), such that mutants could be easily isolated by the fifth and sixth passage without selection. Chloramphenicol-sensitive clones were screened by PCR to determine whether they contained mutant or revertant alleles (Fig. 4B). Deletion mutants of *cps2E* were isolated at the fifth nonselective passage and confirmed by PCR.

High-frequency allele exchange offers a widely applicable alternative to the use of counterselection markers in the generation of precise, markerless mutations. As the development of conterselection markers can be challenging, this high-frequency approach should be particularly useful in organisms for which counterselection markers have not been established (11). In fact, our findings suggest that there may be less need for counterselection markers than previously envisaged.

## Supplementary Material

Supplemental material
