# Supplementary material for: Improved Bacterial Mutagenesis by High-Frequency Allele Exchange, Demonstrated in Clostridium difficile and Streptococcus suis
Source: Appl Environ Microbiol. 2013 Aug;79(15):4768–71. doi: 10.1128/AEM.01195-13 (PMC3719504; doi:10.1128/AEM.01195-13)
Supplement: Supplemental material [file supp_79_15_4768__index.html]

Improved Bacterial Mutagenesis by High-Frequency Allele Exchange, Demonstrated in Clostridium difficile and Streptococcus suis — Supplemental material 

# Improved Bacterial Mutagenesis by High-Frequency Allele Exchange, Demonstrated in Clostridium difficile and Streptococcus suis

## 

**Files in this Data Supplement:**

- Supplemental file 1 -

  Supplemental materials and methods (MIC assays, identification of suitable suicide vector for *S. suis*); list of ribotype 017 human isolates used in lincomycin/erythromycin MIC assay (Table S1); list of primers (Table S2).

  PDF, 162K
