# Supplementary material for: Improved Bacterial Mutagenesis by High-Frequency Allele Exchange, Demonstrated in Clostridium difficile and Streptococcus suis
Source: Appl Environ Microbiol. 2013 Aug;79(15):4768–71. doi: 10.1128/AEM.01195-13 (PMC3719504; doi:10.1128/AEM.01195-13)
Supplement: Supplemental material [file AEM.01195-13_zam999104576so1.pdf]

Supplementary Materials and Methods

Minimum inhibitory concentration assays

Minimum inhibitory concentration (MIC) assays were performed in 24-well plates as described previously (1); the optical density was measured 16 h post inoculation at λ595 in a Spectrophotometer (Biotek, UK).

Identification of a suitable suicide vector for use in *Streptococcus suis*

The replication defective *Clostridium difficile* vector, pMTL82151, was tested to determine whether it was non-replicative in *S. suis*, and so could be used as a suicide plasmid in this organism. *S. suis* P1/7 competent cells were transformed with pMTL82151 and could not form colonies on chloramphenicol 5 $\mu g.ml^{-1}$ BHI agar, while cells transformed with the replicative plasmid pSET1 (2) formed colonies as expected.

Supplementary Tables

Table S1. List of ribotype 017 human isolates used in lincomycin/ erythromycin minimum inhibitory concentration assay. Date indicates date isolated.

Strain	MLST type	Location	Date
M68	37	Ireland	2006
CF3	37	Belgium	1995
ES173	37	Australia	26.06.2006
CD586	37	UK	11.03.2009
CD637	37	UK	16.04.2009
CD816	37	UK	28.09.2009
CD825	37	UK	01.10.2009
CD839	37	UK	29.10.2009

Table S2. List of primers

Primer name	Sequence (5’-3’)	Function (restriction site)
M68 *ermB* RU	ATTTCCTCCCGTTAAAATTTTTGTTCATGTAA	Amplify M68 *ermB* HA1 (universal)
M68 *ermB* FU	TTACATGAACAAAAATTTTAACGGGAGGAAAT	Amplify M68 *ermB* HA2 (universal)
M68 *ermB* F1 -300	TTATAATGAATTCAATCCAACAAGCAAAATCTG	Amplify M68 *ermB* HA1 - 300 bp (EcoRI)
M68 *ermB* R2 +300	ACTTAGCCTGGATCCCCTCTAGTAATTCTTTTACA	Amplify M68 *ermB* HA2 + 300 bp (BamHI)
M68 *ermB* F1 -600	TTATAATGAATTCAAAAAAAGCGGTTGAATCG	Amplify M68 *ermB* HA1 - 600 bp (EcoRI)
M68 *ermB* R2 +600	AATTAGACTGGATCCAGTATTTTGAGTTCTTCCTT	Amplify M68 *ermB* HA2 + 600 bp (BamHI)
M68 *ermB* F1 -1200	TTGTGATCGAATTCAGAAATAGAAAGAAGTGAGT	Amplify M68 *ermB* HA1 - 1200 bp (EcoRI)
M68 *ermB* R2 +1200	AATTAGACTGGATCCACTAAATAAGGAACAAAACC	Amplify M68 *ermB* HA2 + 1200 bp (BamHI)
M68 *fliC* F1	ATAATATAAGAATTCATAGATAACATTGATTATAA	Amplify M68 *fliC* HA1 - 1200 bp (EcoRI)
M68 *fliC* R1	CTAATAATTGTAAAACATTAACTCTCATTATT	Amplify M68 *fliC* HA1
M68 *fliC* F2	AATAATGAGAGTTAATGTTTTACAATTATTAG	Amplify M68 *fliC* HA1
M68 *fliC* R2	TTATATTGGATCCTAAGCTAAAGTACACATT	Amplify M68 *fliC* HA1 + 1200 bp (BamHI)
630 *fliC* F1	ACTTAGCCTGAGCTCAATTCATTTAAAATAGATAA	Amplify M68 *fliC* HA1 - 1200 bp (SacI)
630 *fliC* R1	AACTCCTTGTGGTTGTTGATTTGTATTAACTCTCAT	Amplify M68 *fliC* HA1
630 *fliC* F2	ATGAGAGTTAATACAAATCAACAACCACAAGGAGTT	Amplify M68 *fliC* HA1
630 *fliC* R2	GCGTGCTAGGATCCTAATTTATATAAAGACATAA	Amplify M68 *fliC* HA1 + 1200 bp (BamHI)
M68Δ*ermB* screen F	GCGAATCATTCAATTAGAAAACG	PCR screen M68Δ*ermB*
M68Δ*ermB* screen R	ATCGTTGTGCTGTCCGTATC	PCR screen M68Δ*ermB*
M68 Δ*fliC* screen F	TAAGTTTTCAATTAGATGAG	PCR screen M68Δ*fliC*
M68 Δ*fliC* screen R	ATGCTGATAACGATTTTAAA	PCR screen M68Δ*fliC*
630 Δ*fliC* screen F	AGTAGTCAATGTAGTCGAAGAT	PCR screen 630 Δ*fliC*
630 Δ*fliC* screen R	GAATTTGGAATCTCTCTATAATAG	PCR screen 630 Δ*fliC*
cps2E RU	TTACTTACTTCCCTCTCTCAATATTTCAATATTCATAGCTCCT	Amplify P1/7 *cps2E* HA1 universal
cps2E FU	AGGAGCTATGAATATTGAAATATTGAGAGAGGGAAGTAAGTAA	Amplify P1/7 *cps2E* HA2 universal
cps2E F -600	TATATTGAATTCAAAATCATCATGATTAACTTTC	Amplify P1/7 *cps2E* HA1 -600 bp (EcoRI)
cps2E R +600	AGTTCAGGATCCTAAGCAATATAAGATGTTTCAG	Amplify P1/7 *cps2E* HA2 +600 bp (BamHI)
cps2E F -1200	TATATTGAATTCAATTACAAAGATTACAGGTTTG	Amplify P1/7 *cps2E* HA1 -1200 bp (EcoRI)
cps2E R +1200	AGTTCAGGATCCTCCTTTAAACAACTTCTCATAC	Amplify P1/7 *cps2E* HA1 +1200 bp (BamHI)
cps2E screen F	CTGCGGCTAGTCTCGCTATT	PCR screen P1/7 Δ*cps2E*
cps2E screen R	CATGCGCTTCAAATTCATTC	PCR screen P1/7 Δ*cps2E*
M13F	ACTGGCCGTCGTTTTACA	PCR screen
M13R	CAGGAAACAGCTATGACC	PCR screen

HA = homology arm; bp = base pairs; F = Forward; R = Reverse. Underlined sequences correspond to recognition sequences for restriction endonucleases

References

1. **Andrews, J. M.** 2001. Determination of minimum inhibitory concentrations. J Antimicrob Chemother **48 Suppl 1:**5-16.
2. **Takamatsu, D., M. Osaki, and T. Sekizaki.** 2001. Construction and characterization of *Streptococcus suis-Escherichia coli* shuttle cloning vectors. Plasmid **45:**101-113.
